# Dissecting Alzheimer's disease heritability across populations

**DOI:** 10.1002/alz.71236

**Published:** 2026-03-25

**Authors:** Shiying Liu, William S. Bush, Brian W. Kunkle, Goldie S. Byrd, Christiane Reitz, Giuseppe Tosto, Farid Rajabli, Allison M. Caban‐Holt, Michael Cuccaro, Takiyah Starks, Larry D. Adams, Briseida E. Feliciano, Rufus Akinyemi, Jeffery M. Vance, Margaret Pericak‐Vance, Jonathan L. Haines, Dana C. Crawford, Scott M. Williams

**Affiliations:** ^1^ Department of Population and Quantitative Health Sciences Case Western Reserve University School of Medicine Cleveland Ohio USA; ^2^ John P. Hussman Institute for Human Genomics Dr. John T. Macdonald Foundation Department of Human Genetics Department of Neurology University of Miami Miller School of Medicine Miami Florida USA; ^3^ Maya Angelou Center for Health Equity Wake Forest School of Medicine Winston‐Salem North Carolina USA; ^4^ Gertrude H. Sergievsky Center Taub Institute for Research on Alzheimer's Disease and the Aging Brain Department of Neurology Columbia University Department of Epidemiology Columbia University New York New York USA; ^5^ The Taub Institute for Research on Alzheimer's Disease and the Aging Brain Columbia University Irving Medical Center Columbia University; Department of Neurology Columbia University Irving Medical Center Columbia University; The Gertrude H. Sergievsky Center College of Physicians and Surgeons Columbia University Irving Medical Center Columbia University New York New York USA; ^6^ Universidad Central del Caribe Bayamón Puerto Rico USA; ^7^ Neuroscience and Ageing Research Unit College of Medicine University of Ibadan Ibadan Nigeria

**Keywords:** *APOE* ε4, cohort, family‐based, heritability, multiple populations

## Abstract

**INTRODUCTION:**

Late‐onset Alzheimer's disease (LOAD) is highly heritable; however, its estimated incidence across populations remains unclear.

**METHODS:**

We computed family‐based heritability leveraging Alzheimer's Disease Sequencing Project pedigrees from non‐Hispanic White (404 pedigrees), non‐Hispanic Black (13 pedigrees), Dominican (100 pedigrees), and Dutch isolate (10 pedigrees), with four models incorporating age, sex, apolipoproten E epsilon4 (*APOE* ε4), and contributing study using two methods.

**RESULTS:**

Heritability estimates varied by method, model, and study populations. Statistical Analysis for Genetic Epidemiology (S.A.G.E.) estimates were highest for Dutch isolate (78.3%), followed by non‐Hispanic Blacks (39.1%), Dominicans (31.7%), and non‐Hispanic Whites (29.1%), adjusted for age and sex. *APOE* adjustment reduced estimates (4.9% on average), while study adjustment primarily affected groups that included multiple studies. Sequential Oligogenic Linkage Analysis Routines (SOLAR‐Eclipse) estimates were higher (45.2% to 80.2%) than S.A.G.E. (20.4% to 80.9%) but behaved in parallel, except for the Dutch isolate.

**DISCUSSION:**

LOAD heritability estimates are dependent on study population and may reflect or indicate differences in LOAD risk by population.

## Highlights

- LOAD heritability estimates varied by method, model, and populations.
- By population: Dutch isolate > non‐Hispanic Blacks > Dominicans > non‐Hispanic Whites.
- Models for Puerto Ricans failed to converge due to sample size.
- *APOE* adjustment reduced estimates varied by population (4.9% on average).
- *S*tudy adjustment primarily affected groups that included multiple studies.

## 1 BACKGROUND

Late‐onset Alzheimer's disease (LOAD) is a devastating neurodegenerative disorder that affects people aged 65 years or older, severely impairing cognitive function and quality of life. Estimated to affect over 6.9 million individuals in the United States, LOAD represents one of the most pressing public health challenges as prevalence is predicted to increase substantially due to more people entering high‐risk age groups.^1^ Beyond the profound impact on the affected individuals, LOAD imposes substantial financial and caregiving burdens on families and healthcare systems worldwide.^2^, ^3^ In contrast to early‐onset Alzheimer's disease (AD) that is often linked to autosomal dominant mutations, LOAD is a complex disorder influenced by the interplay of multiple genetic and environmental factors.^4^, ^5^ Decades of research have established that genetic variation contributes to LOAD pathogenesis, with familial aggregation studies demonstrating an increased risk among those with relatives of affected individuals.^6^ In the Swedish Twin Registry, twin studies have estimated LOAD heritability, defined as the proportion of phenotypic variance due to genetics,^7^ can be as high as 79%.^8^ However, heritability estimates have been estimated mostly in populations of European ancestry and the potential variability across populations is unknown, leading to a critical gap in our understanding of the genetic underpinnings of LOAD in non‐European populations.

Variability in the underlying etiology of LOAD is supported by several lines of evidence, including the substantial variation in prevalence across racial and ethnic groups. Both African Americans and Hispanics have a significantly higher prevalence of LOAD compared to non‐Hispanic Whites. Asian populations have a wide range of disease burden.^9^, ^10^, ^11^, ^12^ This variation underscores the need to investigate population‐specific genetic and environmental risk factors for LOAD. At the genetic level, the ε4 allele of the apolipoprotein E (*APOE*) gene is the most consistent and strongest genetic risk factor for LOAD,^13^ but its effect size varies significantly among populations; East Asians have the largest odds ratios conferred by ε4, with either African Americans or Hispanics having the lowest.^14^, ^15^, ^16^ The lack of comprehensive studies across populations has hindered efforts to fully elucidate these and other genetic differences that can be captured by these population descriptors, thereby impeding a comprehensive understanding of pathophysiology and development of tailored interventions.

Despite numerous heritability studies of LOAD, its precise genetic architecture remains unclear. While the twin‐based heritability estimates have historically been the standard for heritability estimation, their fundamental assumption regarding the equal shared environments between the monozygotic and dizygotic twin pairs often does not reflect real‐world settings where higher similarities both in shared environments and lifestyle have been observed among monozygotic twins.^17^, ^18^, ^19^ Moreover, their reliance on only a few twin population cohorts limits the generalizability of findings. Advances in genome‐wide association studies (GWASs) have enabled single‐nucleotide polymorphism (SNP)‐based heritability estimation,^20^ but SNP‐based estimates typically yield lower estimates than those derived from twin studies.^21^, ^22^ This discrepancy underscores the potential limitations of SNP‐based heritability estimates and highlights the need for alternative approaches to assess genetic contributions to LOAD.^23^ Furthermore, the relative lack of representation of non‐European populations in genetic studies limits broad applicability of these findings.^24^ Family‐based studies offer a powerful methodology for heritability estimation, as they can capture genetic contributions beyond common SNPs used for SNP‐based heritability.^25^ Family‐based studies are particularly valuable for examining LOAD in multiple populations, where genetic architecture and environmental factors can differ substantially.

Our aim was to examine the LOAD heritability across multiple populations from which extensive pedigree data were available and to evaluate the relative contribution of critical factors, such as *APOE* ε4 allele carriage, recorded race/ethnicity, and study‐specific effects. Using families from four racial and ethnic groups, we estimated heritability using four models incorporating different combinations of covariates: (1) age and sex, (2) age, sex, and *APOE* ε4 carrier status, (3) age, sex, and contributing studies, and (4) age, sex, *APOE* ε4 carrier status, and contributing studies. We also sought to gain insights into variation in heritability estimations due to analytical methods and their interpretation by employing two widely used software packages, Statistical Analysis for Genetic Epidemiology (S.A.G.E.) and Sequential Oligogenic Linkage Analysis Routines (SOLAR‐Eclipse). By addressing these objectives, we provide a comprehensive analysis regarding the genetic architecture of LOAD, particularly in under‐studied populations, highlighting the population‐ and methodology‐specific nature of heritability estimates that should be interpreted in the context of study design and family structure.

## 2 METHODS

### 2.1 Study participants

Participants were ascertained as part of the Alzheimer's Disease Sequencing Project (ADSP) family study design component. Initiated in 2012, ADSP is an ongoing study investigating the genetic underpinnings of AD and related dementias across diverse populations. The first three phases of ADSP included discovery, discovery extension, and a follow‐up study. In 2023, ADSP entered the follow‐up study 2.0 diversity initiative phase, incorporating 77 contributing studies with genetic data and other critical measurements.

**RESEARCH IN CONTEXT**

1. **Systematic review**: The authors conducted a literature review of AD heritability primarily through PubMed, along with conference presentations. While previous twin‐based and genome‐wide heritability studies established a substantial role of genetics in LOAD, the studies were predominantly conducted in people of European ancestry, thereby limiting insights into the genetic architecture across different populations.
2. **Interpretation**: Our findings demonstrate that family‐based heritability estimates of LOAD vary substantially across populations, but with consistent trends observed using different model configurations. Critical factors, including *APOE* ε4 and contributing study, affected observed heritability estimates. Our results indicate that population‐specific factors, including unmeasured environmental factors, need to be considered in defining the genetic risk and, therefore, mitigating risk.
3. **Future directions**: This study focuses on family‐based heritability across populations, but SNP‐based heritability using overlapping participants is an essential extension of this work to enable direct comparisons and explore missing heritability. Additional insight will also come from larger sample sizes, particularly from underrepresented groups. Deeper phenotyping and more complete family structure data will also be useful in elucidating the genetic architecture of LOAD.

The present study focuses on the family‐based samples from the most recent release of the ADSP genotype family‐based data, which prioritized ethnically diverse populations while excluding families with established Mendelian mutations for AD.^26^ Multiplex families were ascertained from diverse contributing studies and further screened for inclusion by requiring each included pedigree to have at least two members with LOAD and available *APOE* genotypes while excluding families having Mendelian mutations for LOAD or non‐AD dementia.^26^ Included in our study is the family subset of the ADSP R4 release comprising 12,858 participants from nine contributing studies. Each individual included had the following data: demographics (age, sex, race, ethnicity), *APOE* status, LOAD determination, contributing study, and group assignment (defined as family‐level population, race, or ethnicity).

### 2.2 Pedigree inspection

From the initial 12,858 participants, 12,804 were categorized into five distinct group assignments, each designated with a unique value: non‐Hispanic White (*n* = 7,710; 415 pedigrees), non‐Hispanic Black (*n* = 319; 14 pedigrees), Dominican (*n* = 3,988; 100 pedigrees), Dutch isolate (*n* = 655; 10 pedigrees), and Puerto Rican (*n* = 132; three pedigrees). Meanwhile, certain pedigrees had missing group assignments for all individuals within (*n* = 13; three pedigrees) or had multiple assigned values (*n* = 41; one pedigree). To facilitate comparisons of heritability estimates between and across group assignments (e.g., populations), we first inspected individual participant race/ethnicity records within group assignments. We identified 15 out of 546 multiplex pedigrees, including 11 pedigrees collected as part of ascertainment efforts focused on non‐Hispanic White families and three collected as part of efforts focused on non‐Hispanic Black families (Table S1).

We identified a total of 49 individuals whose group assignment required manual inspection, and a multistep approach was used to evaluate pedigrees prior to estimating heritability as described below. Within each pedigree, we inspected each participant's race and ethnicity records and specific pedigree structures to determine whether these individuals or pedigrees would be included in the heritability estimation. We then developed pedigree‐specific strategies for inclusion and exclusion, followed by heritability estimation based on the different strategies. We preferentially retained informative individuals and pedigrees and computed a series of preliminary heritability estimates to guide individual and pedigree selection for the final dataset as described below.

**FIGURE 1 alz71236-fig-0001:**
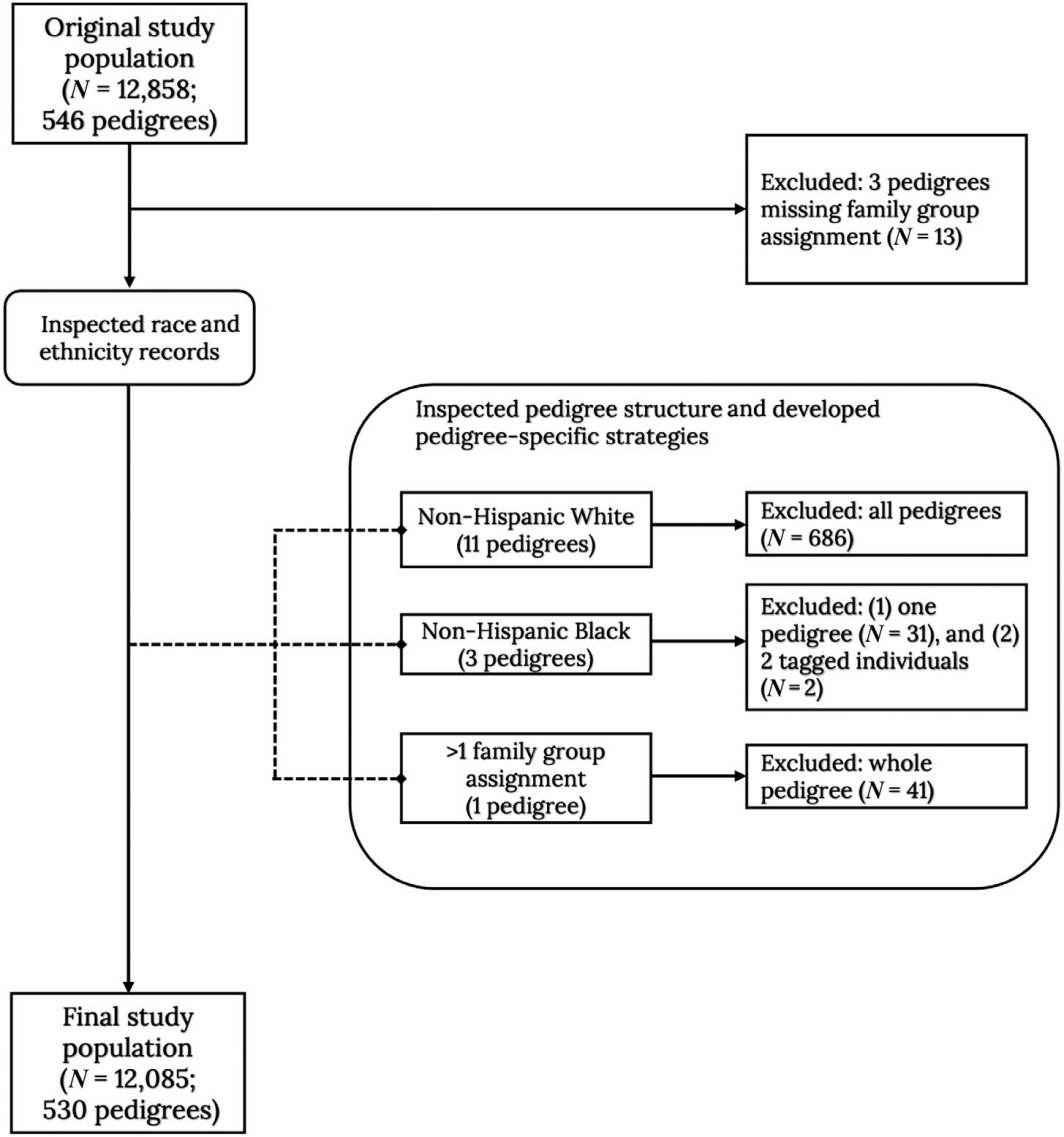
Pedigree inspection to create final study population for analysis.

Based on inspection (Figure S1), we finalized our study participants by removing all 11 flagged pedigrees from the non‐Hispanic White group and removing one pedigree with more than one group assignment. For the non‐Hispanic Black group, one pedigree with two non‐African American members and missing LOAD status were completely removed, while for the other two flagged pedigrees, we only removed individual non‐African American members and retained the remaining pedigree members for heritability analysis (Figure 1). In addition to the inspection, we also examined pedigree structure along with effective sample size and determined that the Puerto Rican group, with only 132 members across three pedigrees, was too small for downstream analyses. The final set of participants in the subsequent analysis included four distinct groups with 11,953 individuals in 527 pedigrees (Table 1).

**TABLE 1:** Sample sizes and demographics, by group assignment.

	Non‐Hispanic White	Non‐Hispanic Black	Dominican	Dutch	*p* value
**No. individuals**	7,024	286	3,988	655	
**No. pedigrees**	404	13	100	10	
**AD = Affected (%)**	1,088 (41.2)	33 (39.3)	600 (47.1)	60 (36.4)	0.002
**Age**	70.79 (12.38)	66.89 (12.48)	68.85 (11.01)	78.36 (7.20)	<0.001
Age of onset for affected participants	77.05 (8.22)	76.18 (7.30)	74.15 (9.15)	73.55 (8.25)	
Age at last assessment for unaffected participants	65.78 (13.16)	59.63 (10.55)	64.25 (10.16)	81.10 (4.70)	
**Sex = Female (%)**	3,663 (52.1)	147 (51.4)	2,062 (51.7)	361 (55.1)	0.442
**Race (%)**					
American Indian/Alaska Native	0 (0.0)	0 (0.0)	1 (0.1)	0 (0.0)	
Black or African American	0 (0.0)	149 (100.0)	82 (5.5)	0 (0.0)	
White	4,500 (100.0)	0 (0.0)	453 (30.3)	366 (100.0)	
Other	0 (0.0)	0 (0.0)	961 (64.2)	0 (0.0)	
**Ethnicity = Hispanic (%)**	0 (0.0)	0 (0.0)	1,497 (100.0)	0 (0.0)	
***APOE* ε4 Carrier = Yes (%)**	1,468 (56.1)	49 (48.0)	509 (39.1)	73 (45.3)	<0.001

*Note*: Shown are the sample size and demographics for individuals from Alzheimer's Disease Sequencing Project (ADSP). Age represents age at onset for cases and age at last examination or family report for controls. Note that the percentages presented in the table were based on participants with complete data for the corresponding variables. AD status, age, sex, race, ethnicity, and *APOE* ε4 carrier status (at least one copy) were compared using Fisher's exact and two‐tailed *t*‐tests, where appropriate.

Abbreviations: AD, Alzheimer's disease; APOE, apolipoprotein E.

### 2.3 AD phenotyping and covariates

#### 2.3.1 Phenotyping determination

The participants were evaluated using the National Institute of Neurological Disorders and Stroke–Alzheimer's Disease and Related Disorders Association (NINDS‐ADRDA) criteria.^26^, ^27^ AD status includes possible designations of no dementia, definite AD, probable AD, possible AD, family reported AD, family reported no AD, and unknown. In this study, LOAD cases were defined as individuals having definite, probable, or possible AD diagnoses (*n* = 1,798), while the unaffected controls were those without clinical dementia at their most recent visit (*n* = 2,408). Participants with family reported AD, family reported no AD, and unknown AD status were coded as missing for LOAD status in the following analyses (*n* = 7,879).

#### 2.3.2 Age

Age was determined as the age of onset for LOAD cases and as the age at the last assessment for the unaffected controls. Meanwhile, for privacy protection and continuous variable consistency, individuals with an age greater than 90 were recorded as 90 in all analyses.

#### 2.3.3 *APOE* ε4 carrier status

In the data we accessed, *APOE* genotypes were available either by direct genotyping or whole‐genome sequencing. A certain proportion (7.7%) had genotypes from both methods. When discordant (*n* = 7), whole‐genome sequencing‐derived *APOE* genotypes were used (Table S2). For analyses, we created a binary variable indicating the presence of at least one copy of *APOE* ε4 allele to represent *APOE* ε4 carrier status.[Fig alz71236-fig-0001]

### 2.4 Statistical analysis

Leveraging the variance component approach, the total phenotypic variance (σ^2^) can be partitioned into genetic variance (σ^2^_G_) and environmental components (σ^2^_E_), with the former further divided into the additive genetic variance (σ^2^_g_), dominance variance, and epistatic variance. The narrow‐sense heritability (*h* ^2^) is determined as total phenotype variance explained by the additive genetic variance ($h^{2}=\frac{\sigma_{g}^{2}}{\sigma^{2}}\;$). To assess the impact of key variables on heritability estimates, we constructed four models with different covariate adjustments: (1) age and sex, (2) age, sex, and *APOE* ε4 carrier status, (3) age, sex, and contributing study, and (4) age, sex, *APOE* ε4 carrier status, and contributing study. As there were varying levels of missingness for these variables, the analyses were performed focusing on participants with complete data for each model configuration.

Our primary analysis employed the ASSOC program within the S.A.G.E. software package.^28^ ASSOC utilizes a multiple linear regression model leveraging familial correlations that partitions total residual variance into individual components and uses maximum likelihood estimation to determine variance components and covariate coefficients simultaneously. In parallel, we computed heritability estimates using the Sequential Oligogenic Linkage Analysis Routines (SOLAR‐Eclipse) software package^29^ for the same set of participants focusing on the four models mentioned earlier. This dual‐method approach allowed us to examine the consistency and variation of heritability estimates across these two commonly used methods and to further access the sensitivity of results to methodological differences. SOLAR‐Eclipse implements a maximum‐likelihood variance‐components approach, using a mixed‐effects model incorporating fixed covariate effects, additive genetic effects, and residual error to estimate polygenic heritability. Notably, for binary traits such as LOAD status, both S.A.G.E. and SOLAR implement a liability threshold model that assumes an underlying latent continuous liability distribution with a threshold to determine the binary status of the trait. The 95% confidence intervals (CIs) were computed based on the estimates along with standard errors and truncated at the theoretical bonds of heritability (0 to 1). We also compared the heritability estimates across groups using pairwise *t*‐tests leveraging the corresponding standard errors. Basic analyses and file processing were conducted using R version 4.4.1.^30^

## 3 RESULTS

### 3.1 Description of participants

After pedigree inspection and quality control, our final study population consisted of 11,953 participants across four group assignments (Table 1), among whom 1,106 were sequenced (9.3%). The study sample had an average mean age of 70.43 years, was predominantly female (52.1%), and comprised approximately 50% *APOE* ε4 allele carriers based on available data on *APOE*. Age distributions varied significantly among group assignments, with the Dutch isolate showing the highest mean age of onset or last assessment (78.36 years) and non‐Hispanic Black participants showing the lowest (66.89 years). Furthermore, except for the Dutch isolate group, the age of last assessment for controls was much younger than the age of onset for affected individuals across the three other groups, especially for the non‐Hispanic Black group, where the mean age of last assessment (59.63 years) was far below the age of onset for LOAD, raising the possibility that some currently classified as unaffected may develop AD in the future, potentially leading to phenotypic misclassification and subsequent underestimation of heritability. As expected, the proportion of AD cases differed across groups, largely exceeding population‐specific prevalence rates for their respective race and ethnicity groups.^1^ Additionally, the number of affected individuals was not uniformly distributed across groups (Table S3). Overall, families with at least five affected individuals represented the majority across all groups, while the non‐Hispanic White group predominantly had two affected cases per pedigree, reflecting variation in the effective pedigree structure. *APOE* ε4 carrier status also varied substantially across groups, ranging from 56.1% in non‐Hispanic Whites to 39.1% in Dominicans.

### 3.2 The resulting heritability estimates are highly variable

**FIGURE 2:**
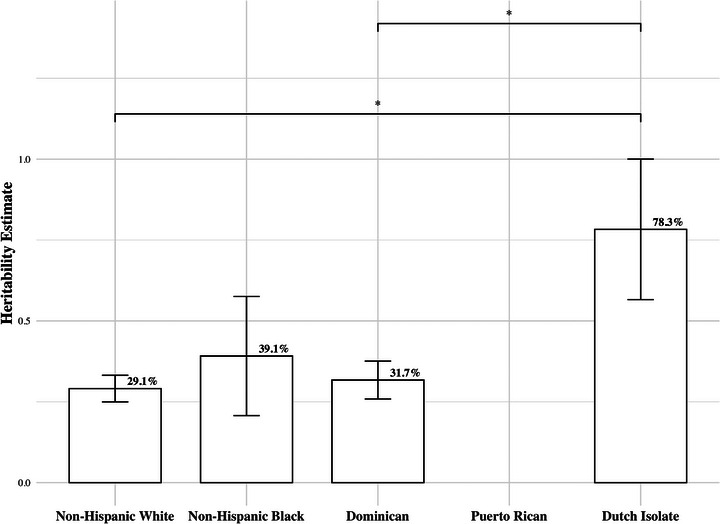
S.A.G.E.‐derived late‐onset Alzheimer's disease heritability estimates across all four group assignments, adjusted for age and sex. Included are individuals from non‐Hispanic White (414 pedigrees; *n* = 2602), non‐Hispanic Black (13 pedigrees; *n* = 84), Dominican (100 pedigrees; *n* = 1271), and Dutch Isolate (10 pedigrees; *n* = 165) group assignments with complete information regarding AD status, age, and sex. In the figure, different group assignments are shown on the *x*‐axis, with the *y*‐axis representing heritability estimates ranging from 0 to 1. Heritability estimates are depicted in bars with bars indicating corresponding standard error. Heritability estimates across groups were compared using pairwise *t*‐test leveraging the corresponding standard errors.

Leveraging S.A.G.E., LOAD heritability estimates under the base model (Model 1, age and sex) demonstrated variation across group assignments, reflecting variation among the sampled multiplex families. Based on pairwise comparisons, the heritability estimate for the Dutch isolates was significantly higher than both the non‐Hispanic Whites and Dominicans (Figure 2). Although statistical significance was borderline (*α* = 0.05), a consistent pattern emerged, with Dutch isolates showing the highest heritability (*h* ^2^ = 78.3%, 95% CI [35.7% to 1]), followed by non‐Hispanic Black (*h* ^2^ = 39.1%, 95% CI [3.0% to 75.3%]), Dominican (*h* ^2^ = 31.7%, 95% CI [20.3% to 43.2%]), and non‐Hispanic White (*h* ^2^ = 29.1%, 95% CI [21.0% to 37.1%]) populations (Figure 2).

**FIGURE 3:**
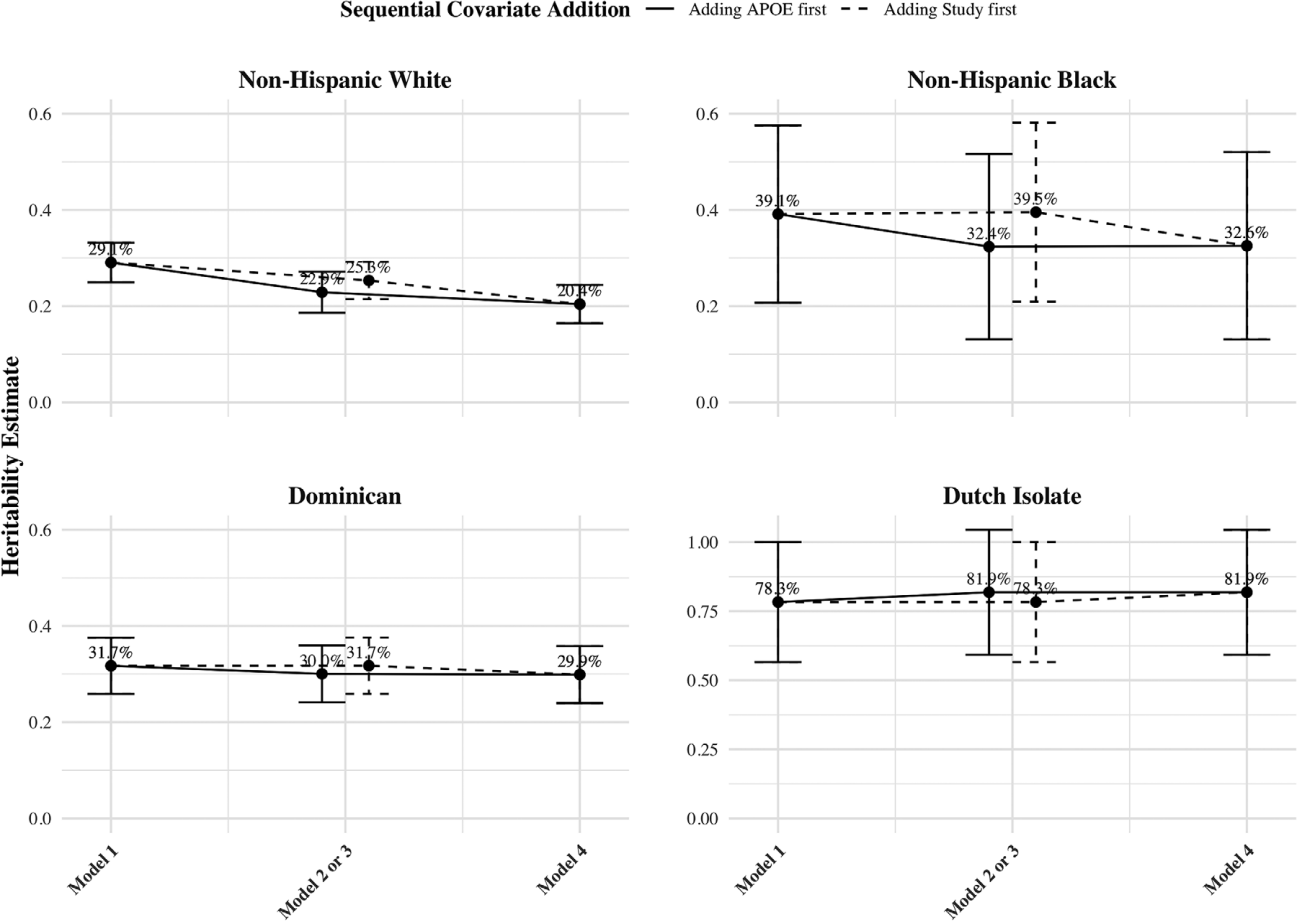
S.A.G.E.‐derived late‐onset Alzheimer's disease heritability estimates by group assignment under four covariate adjustment models. Two sets of sequential covariate addition are shown. One begins with the base model (age and sex), adds *APOE* ε4 carrier status, and concludes with the full model, as represented by solid lines. The other starts with the same base model, adds contributing study effect, and ends with the same full model, as indicated by dashed lines. In each plot, different models are shown on the *x*‐axis. Notably, the *y*‐axis for heritability estimates in Dutch Isolates ranges from 0 to 1, whereas for the remaining group assignments, the *y*‐axis threshold is 0.6 to center within the range for better visualization. Each point represents a heritability estimate with corresponding standard error bars. Model specifications: Model 1 (base): age and sex; Model 2: base + *APOE* ε4; Model 3: base + contributing study; Model 4 (full): base + *APOE* ε4 + contributing study.

Similar patterns were observed across group assignments from models with the addition of covariate adjustments (Models 2 to 4). Specifically, LOAD heritability estimates for the Dutch isolates were consistently higher than non‐Hispanic Black, Dominican, and non‐Hispanic White populations for all models analyzed when adding *APOE* ε4 carrier status (Model 2), the contributing studies (Model 3) or both (Model 4) (Figure 3).

### 3.3 Impact of *APOE* ε4 alleles

To investigate the impact introduced by *APOE*, we examined changes in LOAD heritability estimates following the inclusion of *APOE* ε4 carrier status as a covariate. Using S.A.G.E., the addition of *APOE* ε4 carrier status (Model 2) generally reduced heritability estimates compared to the base model, with one exception: The Dutch isolates had an increase in their heritability estimate (Figure 3). The average reduction in heritability was 4.9%, with non‐Hispanic Blacks (6.7%) and non‐Hispanic Whites showing the largest decreases (6.2%).

This reduction pattern associated with *APOE* was also evident when comparing Model 4 to Model 3, where *APOE* ε4 carrier status was added to a model already adjusted for age, sex, and contributing study effects. The reduction trend was identical to that discussed earlier, with non‐Hispanic Black and non‐Hispanic White group assignments showing the largest decreases (7.0% and 4.9%, respectively), while Dutch isolates again showed an increase in heritability.

### 3.4 Impact of contributing studies

**FIGURE 4:**
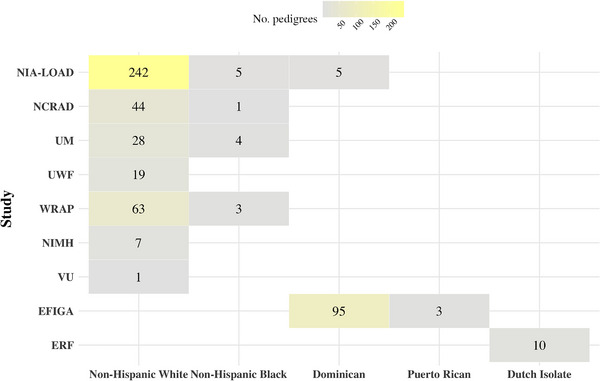
Contributing study distribution of pedigrees, by group assignment. On the *x*‐axis, different group assignments are shown, with contributing studies displayed on the *y*‐axis. Numbers of pedigrees from each contributing study within each group assignment are labeled on color‐coded squares while those without color‐coded squares indicate no individuals from the corresponding study within that specific group assignment. The color intensity corresponds to sample size, with darker yellow indicating larger numbers of pedigrees. NIA‐LOAD, National Institute on Aging Late Onset Alzheimer's Disease Family Study; EFIGA, Estudio Familiar de la Influencia Genetica en Alzheimer; NCRAD, National Cell Repository for Alzheimer's Disease; ERF, Erasmus Rucphen Family; UM, University of Miami; UWF, University of Washington Families; WRAP, Wisconsin Registry for Alzheimer's Prevention; NIMH, National Institute of Mental Health; VU, Vanderbilt University.

We then evaluated the impact of contributing study effects on LOAD heritability estimates, given that participants were ascertained by multiple studies with varying distributions of studies observed across populations (Figure 4). Among those population samples with more than one contributing study, we identified significant differences in age and *APOE* ε4 carrier status across studies for both non‐Hispanic White and non‐Hispanic Black populations that was not evident for Dominicans (Tables S4, S5, and S6). The proportion of females also varied significantly across non‐Hispanic White studies (Table S4), which is particularly noteworthy given known sex differences in LOAD risk.^31^, ^32^ The addition of contributing study as a covariate showed substantial effects, particularly in populations with multiple contributing studies. Non‐Hispanic Whites and non‐Hispanic Blacks, which had the most complicated study compositions, demonstrated the largest changes: a 3.7% decrease and 0.4% increase in heritability, respectively, compared to the base model (Model 1), though these changes were not statistically significant. The changes introduced by the contributing studies were attenuated when comparing Model 4 to Model 2, where *APOE* ε4 carrier status was already included in the reference model (Figure 3). In contrast, the Dominicans and the Dutch isolates, ascertained respectively for only two studies and one study, showed minimal changes in heritability estimates after study adjustment.

### 3.5 Transformed liability‐scale heritability

**FIGURE 5:**
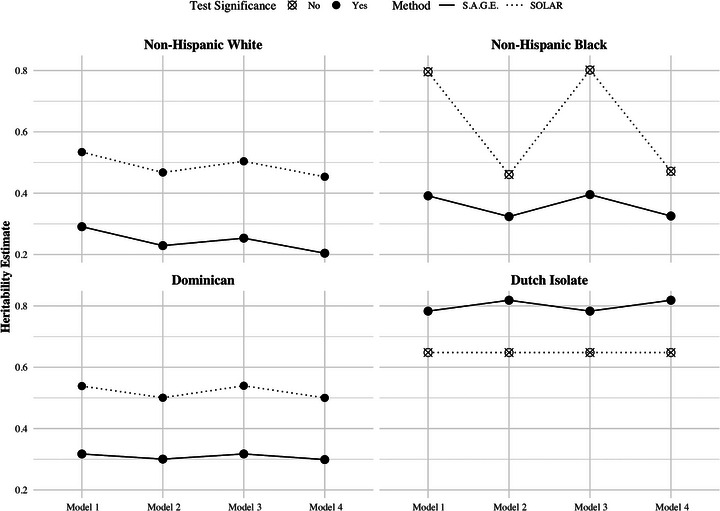
Comparison of S.A.G.E.‐ and SOLAR‐derived heritability estimates across four models by group assignment. Covariates for adjustment in each model: Model 1, age, and sex; Model 2, age, sex, and *APOE* e4 carrier status; Model 3, age, sex, and study; Model 4, age, sex, *APOE ε4* carrier status, and study. In each plot, different models are shown on *x*‐axis, with *y*‐axis representing heritability estimates ranging from 0 to 1. Heritability estimates are shown as points, with solid circles indicating statistically significant findings and crossed circles indicating statistically non‐significant findings from the estimation process. S.A.G.E.‐derived estimates are connected by solid lines, while SOLAR‐derived estimates are connected by dotted lines.

Given that LOAD status is a binary trait, converting heritability estimates to liability scale is crucial for accurate interpretation and comparison across groups.^33^, ^34^ Unlike quantitative traits, a continuous liability threshold model is required, where the threshold inference is based on the prevalence of the trait, to derive the proportion of liability variation explained by genetic components for the binary traits.^35^ We applied a transformation formula adapted from Lee et al., originally developed for SNP‐based heritability,^36^ to obtain corresponding liability‐scale heritability across populations. To do this, we incorporated the population‐specific prevalence (K) using values from Matthews et al.^10^: 10.3% for non‐Hispanic White, 13.8% for non‐Hispanic Black, and 12.2% for Hispanics, respectively, along with the proportion of cases in the study sample (P) as presented in Table 1. The use of transformed heritability estimates on the liability scale maintained consistent patterns across populations and modeling approaches (Figures S2 and S3) compared with our observed scale heritability estimates (Figure 5).

$${\hat{h}}_{l}^{2}={\hat{h}}_{o}^{2}\;\times \frac{K\left(1-K\right)}{z^{2}}\frac{K\left(1-K\right)}{P\left(1-P\right)}$$

### 3.6 Comparison between S.A.G.E. and SOLAR estimates

We conducted parallel analyses using SOLAR with the same model construction in each population or group assignment as S.A.G.E. The SOLAR‐derived heritability estimates generally differed from S.A.G.E. results across multiple models. All populations showed higher heritability estimates with SOLAR (45.4% to 80.2%) compared to S.A.G.E. (20.4% to 81.9%), except the Dutch isolates, where S.A.G.E. estimates were all higher (Figure 5).

The population‐specific patterns remained consistent across these two approaches, with both approaches identifying higher heritability in Dutch isolates and non‐Hispanic Blacks, followed by Dominicans and non‐Hispanic Whites within each model. However, SOLAR‐derived heritability estimates were not statistically significant for the Dutch isolates and non‐Hispanic Blacks in any of the four models. Compared with S.A.G.E., these results may be due to the small sample sizes and lack of sufficient information provided by the pedigrees required by SOLAR. We have different levels of missingness across LOAD phenotype and covariates for each group, thereby affecting the number of individuals and pedigrees included and, in turn, the informative relative pairs available for analysis. For example, in non‐Hispanic White Model 1, S.A.G.E. analyzed 2602 individuals compared to the sample size of 2608 used in SOLAR (Table S7). The methods also differed in pedigree inclusion. SOLAR inspects the pedigree structure as well as missing data to determine the final informative samples, potentially removing pedigrees when missing values affect the valid pedigree structure needed for analysis. Meanwhile, using the same input pedigree data, the pedigree same size could increase with S.A.G.E. due to the inclusion of disjoint pedigrees if missingness leads to smaller connected groups.^29^ In our study, for Models 2 and 4 using non‐Hispanic White, S.A.G.E. used 414 pedigrees, while SOLAR included 403. Conversely, in Dominicans, SOLAR analyzed more pedigrees (103) compared with S.A.G.E. (100) (Table S7). The effective sample sizes, along with the impact on the specific pedigree structure, could introduce bias into the heritability estimates obtained. Despite differences in magnitude, the patterns of change across covariate adjustment models observed in SOLAR were largely similar to those observed in S.A.G.E. analyses (Figure 5).

The phenotyping strategy also plays a major role in the heritability estimates obtained in this study. As detailed in the “Methods” section, the affected individuals were determined to have definite, probable, or possible AD diagnoses in this study, with varying numbers of individuals falling within each diagnostic category across groups (Table S8). To further explore the impact of this case definition, sensitivity analyses were performed for cases defined as having definite or probable AD diagnoses for all groups and definite AD diagnoses only for the non‐Hispanic White group, due to the limited number of definite AD diagnoses in other groups. Both S.A.G.E.‐ and SOLAR‐derived heritability estimates using the definite or probable definition were almost always higher than the original definition including all three diagnoses (Tables S9 and S10). When cases were further restricted to definite AD diagnoses only, heritability estimates in non‐Hispanic Whites were even higher across all model configurations (Table S11). The increase in the estimates based on more stringent diagnostic criteria algins with a previous AD heritability study demonstrating higher heritability for cohorts with pathologically confirmed AD diagnosis compared to clinical diagnosis,^37^ further underlining the need for accurate diagnosis for complex disorders like AD and rigorous diagnostic criteria for heritability estimation.

## 4 DISCUSSION

Although family‐based heritability in LOAD has been well‐studied in European‐ancestry participants,^23^ with twin studies reporting estimates of up to 79%,^8^ estimates across ancestries are less well delineated. Our study addresses this gap by using family data derived from multiple diverse group assignments, including non‐Hispanic White, non‐Hispanic Black, Dominican, and Dutch isolate groups, to investigate family‐based LOAD heritability as a means of capturing patterns associated with different populations. Our analyses revealed heterogeneity in LOAD heritability across samples from different populations, ranging from 29.1% in US‐based non‐Hispanic Whites to 78.3% in Dutch isolates after adjusting for age and sex. Including the *APOE* ε4 allele in models resulted in population‐specific reductions, except for the Dutch isolates, highlighting the importance of population‐specific genetic architecture for LOAD and the effect of this allele. Through comprehensive evaluation of the impact introduced by contributing studies and modeling strategies, we demonstrated the importance of population‐specific context and methodological considerations in heritability estimation, providing crucial insights into the genetic basis of LOAD across diverse groups that affect how we analyze each group.

The differences observed for population‐specific LOAD heritability are notable as they may be related to documented differences in prevalence across racial and ethnic groups.^38^, ^39^ We observed a consistent pattern of heritability across populations (non‐Hispanic White < Hispanic < non‐Hispanic Black < Dutch isolates) regardless of model specifications, consistent with the current knowledge of AD prevalence across populations except for the Dutch isolates, who demonstrated high heritability despite lower prevalence.^9^, ^40^, ^41^ Although previous studies highlighted the importance of non‐genetic factors in the disease risk for LOAD,^38^, ^42^, ^43^ our findings shed light on the variation in genetic underpinnings across populations that may affect prevalence. For example, higher heritability estimates observed for the Dutch isolates and non‐Hispanic Blacks may suggest a relatively smaller influence of environmental and social factors, compared to non‐Hispanic Whites from the United States and Dominicans. Our observed heterogeneity in genetic architecture could help disentangle relative contributions of genetic and environmental factors, including gene–environment interactions, to LOAD risk as they pertain to the specific population, but clearly more targeted data will be necessary to address these issues.

Our primary analyses were performed without liability transformation; we also report transformed heritability estimates to facilitate cross‐study comparison, the findings of which remain consistent with the primary findings regarding population‐specific heritability and the effects of key covariates. Previous epidemiological studies showed heterogeneity in LOAD prevalence, especially for Hispanic populations, reflecting diverse cultural and environmental factors, as well as complex admixture patterns, across groups.^44^, ^45^ This heterogeneity makes it problematic to apply the prevalence estimate to the Dominican sample in our study. Precise LOAD prevalence data for the genetically isolated groups, such as the Dutch isolate, are also scarce. Additionally, the age dependency of AD risk and population prevalence estimates that are not directly comparable further complicates the prevalence estimate matching, potentially introducing bias in liability‐scale transformations.^46^ The generally higher values observed in the liability‐scale estimates, compared to those in the observed scale, likely reflect the elevated proportion of LOAD cases in our study, potentially as a consequence of the recruitment strategy to oversample multiplex AD families.^26^

The major genetic risk for LOAD, the *APOE* ε4 allele, shifted heritability estimates in a population‐specific manner. Including *APOE* ε4 carrier status as a covariate resulted in reductions in the heritability estimates across most populations, using both S.A.G.E. and SOLAR. This reduction is expected and aligns with previous studies examining SNP‐based heritability estimates where excluding the *APOE* region had a similar effect.^23^, ^37^ The magnitude of reduction due to *APOE* ε4 adjustment varied across populations, with the largest decrease observed in non‐Hispanic White, followed by non‐Hispanic Black and Dominican populations (Figures 3 and 5). This pattern is concordant with prior findings showing larger *APOE* ε4 effect sizes in non‐Hispanic White compared to non‐Hispanic Black populations, with the lowest effects among Hispanic groups in some studies.^14^, ^16^ Interestingly, the Dutch isolate population exhibited a slight increase in heritability estimates following *APOE* ε4 adjustment, potentially indicating a unique architecture in this isolated population. Adjustment by *APOE* status makes it clear that these genotypes contribute to LOAD heritability, but larger sample sizes are required to inspect heritability when stratified by *APOE* carrier status.

Further attention needs to be given to the impact of contributing study effects on heritability estimates. In line with a previous SNP‐based LOAD heritability study that showed a reduction from 31.9% to 19.0% after adjusting for the study indicator,^47^ we generally observed decreases in heritability estimates when incorporating the contributing study information into analyses (Figures 3 and 5). This reduction is likely explained by underlying study heterogeneity, especially for several key extrinsic LOAD risk factors. Although prior twin and genetic studies have not demonstrated significant heritability differences across age or sex groups,^8^, ^48^, ^49^ previous research highlighted meaningful variation introduced by study effects.^50^, ^51^, ^52^ These study‐specific differences demonstrated by population characteristics may arise from variations in study design, such as differences in recruitment strategies, and evolving phenotyping criteria. Such heterogeneity, along with unmeasured and variable environmental factors, presents challenges for research trying to estimate the genetic components of LOAD in light of trying to increase sample size and diversity.^53^ While larger, more diverse studies are vital for improving statistical power and generalizability, proper adjustment for study effects and other confounders is essential to ensure heritability estimates are robust and reliable.

Differences in the magnitude of LOAD heritability estimates were observed both in comparison to previously published twin studies and between the two methods we employed, emphasizing heritability interpretation requires consideration of context regarding both study population and methodology. Our estimates for non‐Hispanic Whites (29.1% for the base model using S.A.G.E., decreased to 20.4% with full covariate adjustment) were lower than heritability as presented from prior twin studies based on participants of European ancestry (37% to 79%).^8^, ^48^, ^54^, ^55^, ^56^, ^57^, ^58^ These discrepancies could be attributed to fundamental methodological differences between twin‐ and extended pedigree‐based approaches.^19^, ^59^, ^60^ The age‐dependent nature of LOAD also poses challenges to pedigree‐based methods, as age differences across family members can influence heritability estimates. While twin pairs are age‐matched within the twin study design,^61^ age of onset may still be variable; in comparison, larger pedigrees cannot match as well for age.

The deviations between analytical methods were most evident in non‐Hispanic White and Dominican populations, as SOLAR estimates for non‐Hispanic Black and Dutch isolate groups were not statistically significant (Figure 5), possibly due to insufficient, deep pedigree information required for this method. For non‐Hispanic White and Dominican groups, differences in effective sample sizes partially explain variation in heritability estimates between methods, driven at least in part by distinct approaches to handling missing data in relation to the pedigree structure. S.A.G.E. generally eliminated individuals with missing values while preserving their corresponding connected family structure.^28^ SOLAR, in contrast, removes individuals with missing values while retaining resulting singletons.

Methodological differences extend beyond sample size considerations. S.A.G.E. offers flexibility in evaluating additional components such as sibship and marital effects,^28^, ^62^ while SOLAR specializes in analyzing large, complex pedigrees,^29^ particularly evident in our results for non‐Hispanic Black and Dutch isolate groups. Understanding these methodological nuances is necessary for accurate heritability estimation and interpretation, especially in family‐based studies across multiple populations.

The limitations of the study are that sample sizes varied substantially across populations, with some groups having relatively small numbers of both participants and pedigrees, potentially affecting power to detect meaningful differences in heritability estimates. Meanwhile, despite the consistency in patterns across populations, obtaining appropriate population prevalence estimates posed challenges for the liability transformation.^7^, ^63^, ^64^ Another limitation was the family‐based recruitment strategies that may introduce ascertainment bias; for example, the study design specifically excluded families with established Mendelian mutations for AD, possibly limiting representation of the broader population. Missing data also presented a significant challenge, with varying patterns of missingness across study samples reducing effective sample sizes that were handled differently by the two analytical methods, potentially affecting heritability estimates.

Related to the missing data problem is missing race and ethnicity. For example, the 404 non‐Hispanic White pedigrees include 7,024 individuals, with only 4,500 having race or ethnicity data. Race and ethnicity are presumably self‐described; however, pedigrees included come from multiple undocumented collections, making it impossible to verify method of race and ethnicity designation. We did not assume that individuals with missing race or ethnicity data were the same race and ethnicity as other family members in the same pedigree. Together, these factors underscore that our results are best interpreted as heritability within sampled multiplex families that are informative of population‐level patterns.

Our results indicate LOAD heritability cannot be represented by a single value but demonstrates heterogeneity across diverse populations, reflecting both population‐specific genetics and unmeasured population‐specific and individual‐level extrinsic factors, including lifestyle behaviors, environmental exposures, and social determinants of health. The differential impact of *APOE* ε4 on heritability across populations underscores the importance of capturing its population‐specific effects and how these affect the overall genetic architecture of LOAD. Our findings further emphasize that heritability interpretation requires careful consideration of study design, study population, and methodology. While this study focused on family‐based heritability of LOAD, ongoing work comparing SNP‐based heritability in overlapping participants promises to further disentangle genetic and environmental contributions across populations. These efforts reinforce the importance of genetics in LOAD risk and collectively will advance our understanding of LOAD risk, ultimately informing targeted intervention strategies for disease prevention and risk mitigation.

## CONFLICT OF INTEREST STATEMENT

The authors declare no conflicts of interest. Author disclosures are available in the supporting information.

## CONSENT STATEMENT

All human subjects provided consent.

## Supporting information



Supporting Information

Supporting Information

Supporting Information

Supporting Information

Supporting Information

Supporting Information

Supporting Information

Supporting Information

Supporting Information

Supporting Information

Supporting Information

Supporting Information

Supporting Information

Supporting Information

Supporting Information
