# Supplementary material for: Dissecting Alzheimer's disease heritability across populations
Source: Alzheimers Dement. 2026 Mar 25;22(3):e71236. doi: 10.1002/alz.71236 (PMC13093350; doi:10.1002/alz.71236)
Supplement: Supplementary file 2 — Supporting Information [file ALZ-22-e71236-s011.docx]

###
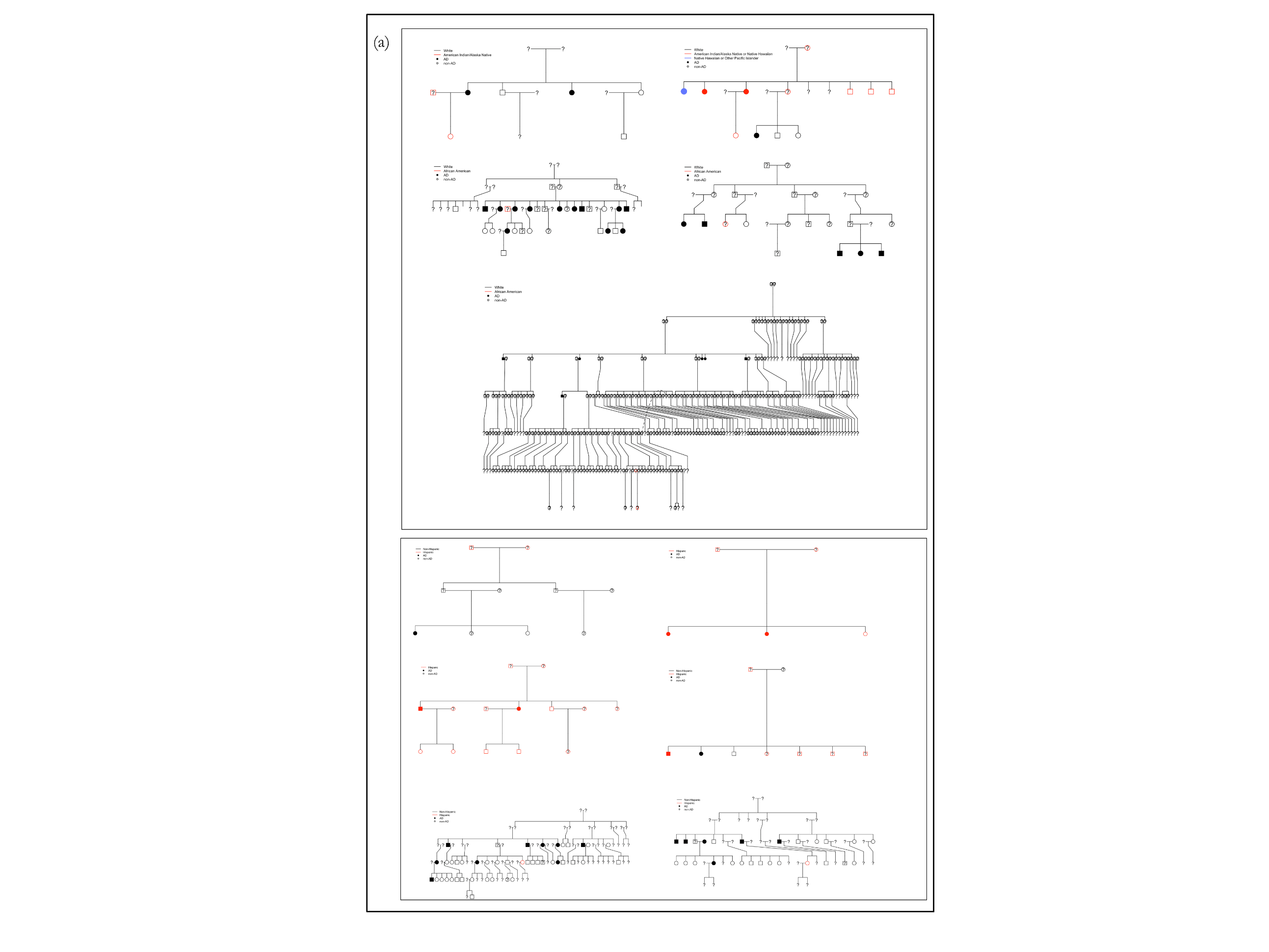


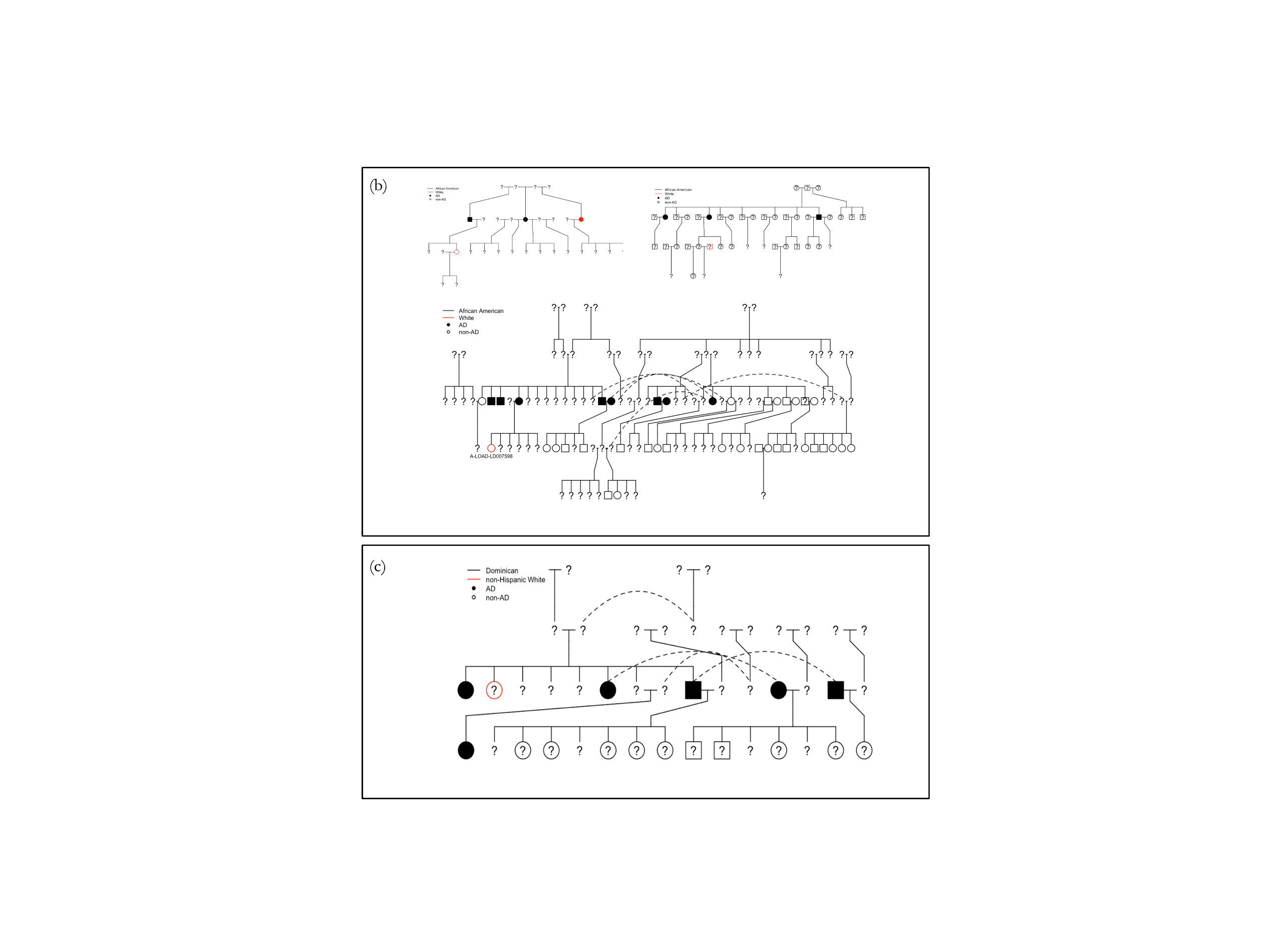


Figure S1 Pedigree inspection for flagged families.

In all pedigrees, circles represent females and squares represent males, with hollow symbols indicating non-AD cases and filled symbols showing AD cases while the missing AD phenotype information is denoted by question marks. The pedigrees are organized into three panels: (a) non-Hispanic White families, (b) non-Hispanic Black families, and (c) family with more than one family group assignments. Color coding varies by these panels: in panel (a), the upper portion uses black to indicate participants labeled as White and red as well as blue for other races, while the lower portion uses black for non-Hispanic and red for Hispanic; in panel (b), black represents participants labelled as African Americans and red indicates other races; in panel (c), black represents participants having the family group assignment of Dominican while red indicates those for non-Hispanic White family group assignment.
