# Supplementary material for: Dissecting Alzheimer's disease heritability across populations
Source: Alzheimers Dement. 2026 Mar 25;22(3):e71236. doi: 10.1002/alz.71236 (PMC13093350; doi:10.1002/alz.71236)
Supplement: Supplementary file 3 — Supporting Information [file ALZ-22-e71236-s008.docx]

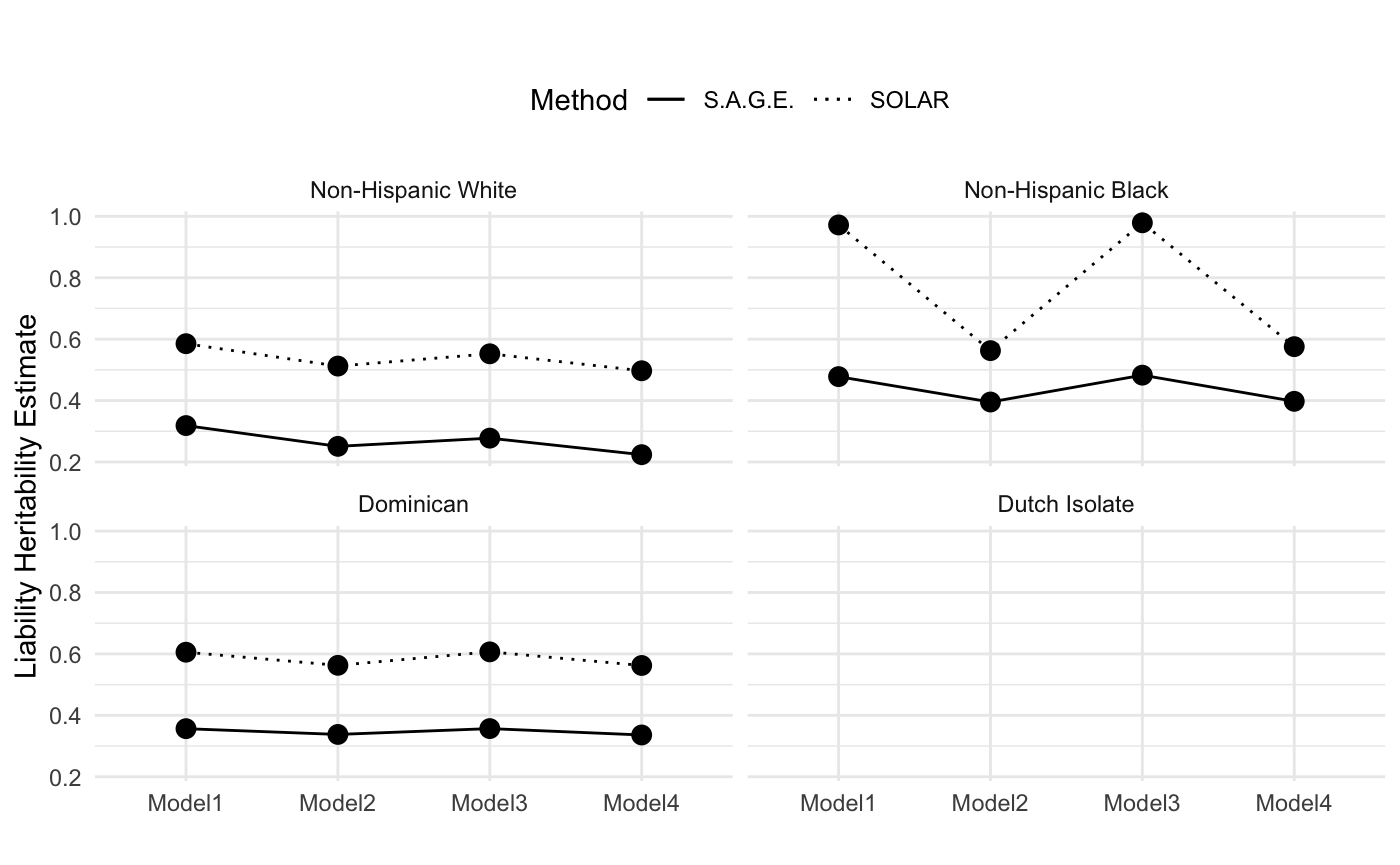


Figure S2 Comparison of S.A.G.E.- and SOLAR-derived heritability estimates transformed to liability scale across four models by family group assignment.

Covariates for adjustment in each model: Model1, age, and sex; Model2, age, sex, and *APOE* e4 carrier status; Model3, age, sex, and study; Model4, age, sex, *APOE* e4 carrier status, and study. In each plot, different models are shown on the x-axis, with the y-axis representing heritability estimates ranging from 0 to 1. Heritability estimates are shown as points. S.A.G.E.-derived estimates are connected by solid lines, while SOLAR-derived estimates are connected by dotted lines. Note that the results for the Dutch Isolates are not shown due to the unavailability of an appropriate population prevalence of LOAD for liability transformation.
