# Supplementary material for: Dissecting Alzheimer's disease heritability across populations
Source: Alzheimers Dement. 2026 Mar 25;22(3):e71236. doi: 10.1002/alz.71236 (PMC13093350; doi:10.1002/alz.71236)
Supplement: Supplementary file 4 — Supporting Information [file ALZ-22-e71236-s005.docx]

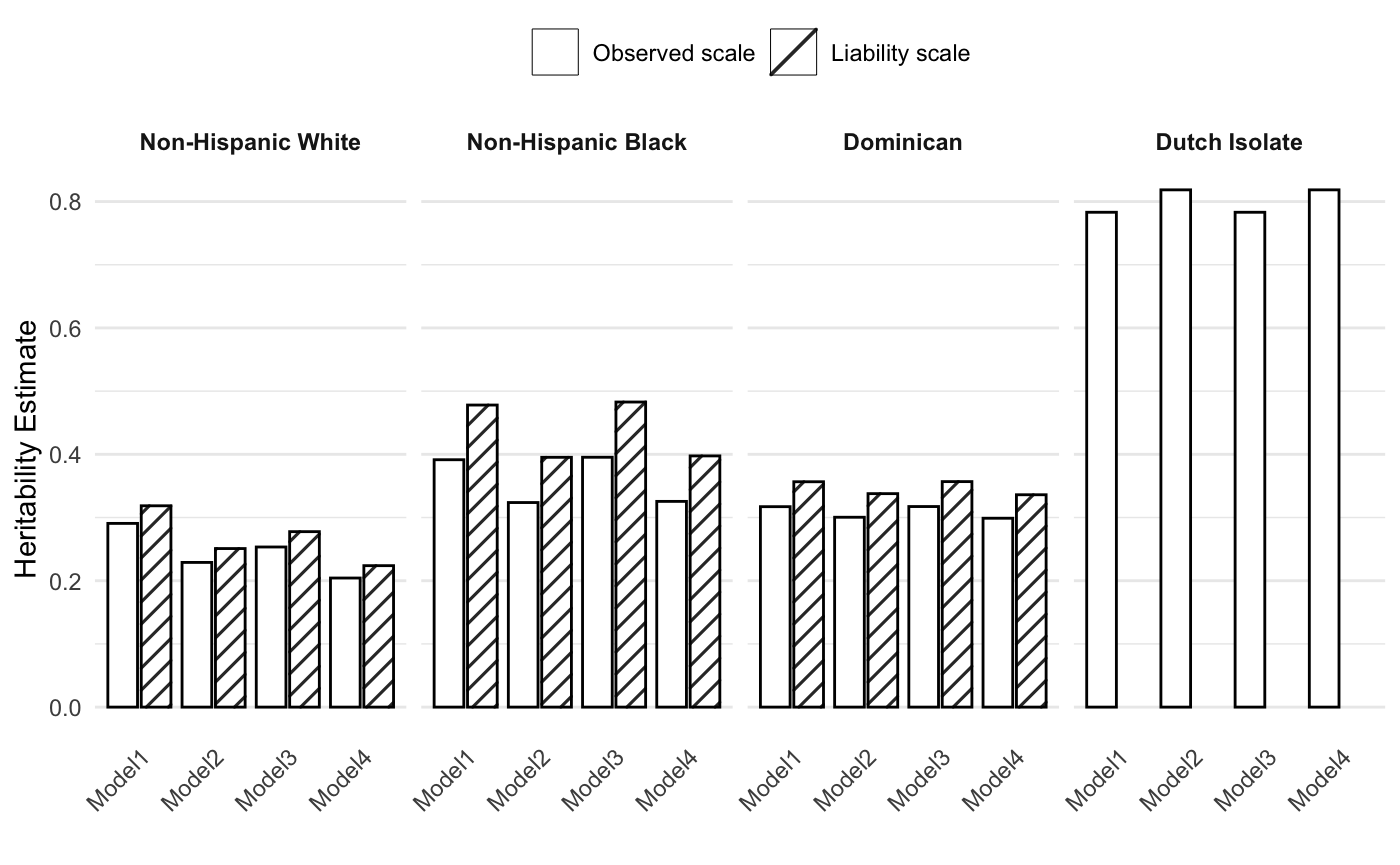


Figure S3 Comparison of S.A.G.E.-derived heritability estimates on both observed and liability scale across four models by family group assignment.

On the x-axis, the findings are grouped into four family group assignments, with each having four models with different covariate adjustments: Model1, age, and sex; Model2, age, sex, and *APOE* e4 carrier status; Model3, age, sex, and study; Model4, age, sex, *APOE* e4 carrier status, and study; with the y-axis representing heritability estimates ranging from 0 to 1. The observed scale heritability estimates are presented by white bars, while the dashed bars represent the heritability estimates transformed to liability scale. Note that the liability scale estimates for the Dutch Isolates are not shown due to the unavailability of an appropriate population prevalence of LOAD for liability transformation.
