# Supplementary material for: Dissecting Alzheimer's disease heritability across populations
Source: Alzheimers Dement. 2026 Mar 25;22(3):e71236. doi: 10.1002/alz.71236 (PMC13093350; doi:10.1002/alz.71236)
Supplement: Supplementary file 5 — Supporting Information [file ALZ-22-e71236-s009.docx]

Table S1 Demographics of pedigrees that upon inspection had individuals of whose group assignment differed from that of the family designation.

|  | **Non-Hispanic White**  (11 pedigrees) | **Non-Hispanic Black**  (3 pedigrees) | **>1 Assignment**  (1 pedigree) |
| --- | --- | --- | --- |
| **n** | 687 | 206 | 41 |
| **AD = Affected (%)** | 53 (40.8) | 13 (28.9) | 4 (100.0) |
| **Age** | 69.42 (14.04) | 64.73 (13.61) | 65.25 (11.44) |
| **Sex = Female (%)** | 321 (46.7) | 97 (47.1) | 25 (61.0) |
| **Race (%)** |  |  |  |
| American Indian/Alaska Native | 10 (2.0) | 1 (1.2) | 0 (0.0) |
| Native Hawaiian or Other Pacific Islander | 1 (0.2) | 0 (0.0) | 0 (0.0) |
| Black or African American | 3 (0.6) | 76 (95.0) | 0 (0.0) |
| White | 478 (97.0) | 2 (2.5) | 8 (53.3) |
| Other | 1 (0.2) | 1 (1.2) | 7 (46.7) |
| **Ethnicity = Hispanic (%)** | 29 (5.7) | 1 (1.2) | 14 (93.3) |
| **Study** |  |  |  |
| NIA-LOAD (%) | 304 (44.3) | 163 (79.1) | 41 (100.0) |
| NCRAD (%) | 383 (55.7) | 43 (20.9) | 0 (0.0) |

The percentages presented in the table were based on participants with complete data for the corresponding variables.
