# Supplementary material for: Dissecting Alzheimer's disease heritability across populations
Source: Alzheimers Dement. 2026 Mar 25;22(3):e71236. doi: 10.1002/alz.71236 (PMC13093350; doi:10.1002/alz.71236)
Supplement: Supplementary file 6 — Supporting Information [file ALZ-22-e71236-s014.docx]

Table S2 Race and ethnicity distribution among participants with discordant *APOE* genotypes between direct genotyping and whole-genome sequencing (n=7).

| **Hispanic**  **Race** | **Yes** | **No** |
| --- | --- | --- |
| **White** | 4 | 2 |
| **Other** | 1 | 0 |
