# Supplementary material for: Dissecting Alzheimer's disease heritability across populations
Source: Alzheimers Dement. 2026 Mar 25;22(3):e71236. doi: 10.1002/alz.71236 (PMC13093350; doi:10.1002/alz.71236)
Supplement: Supplementary file 7 — Supporting Information [file ALZ-22-e71236-s001.docx]

Table S11 Distribution of families by number of affected individuals across group assignment

| # of affected individuals | **Non-Hispanic White** | **Non-Hispanic Black** | **Dominican** | **Dutch Isolate** |
| --- | --- | --- | --- | --- |
| 1 | 23 | 2 | 2 | 0 |
| 2 | 133 | 2 | 6 | 3 |
| 3 | 80 | 5 | 16 | 4 |
| 4 | 48 | 0 | 13 | 0 |
| ≥5 | 60 | 2 | 60 | 3 |
