# Supplementary material for: Dissecting Alzheimer's disease heritability across populations
Source: Alzheimers Dement. 2026 Mar 25;22(3):e71236. doi: 10.1002/alz.71236 (PMC13093350; doi:10.1002/alz.71236)
Supplement: Supplementary file 8 — Supporting Information [file ALZ-22-e71236-s013.docx]

Table S4 Basic demographics across studies for Non-Hispanic Whites.

|  | **NCRAD** | **NIMH** | **NIA-LOAD** | **UM** | **UWF** | **VU** | **WRAP** | **p-value** |
| --- | --- | --- | --- | --- | --- | --- | --- | --- |
| **n** | 899 | 107 | 4643 | 556 | 440 | 6 | 373 |  |
| **AD = A (%)** | 138 (97.9) | 19 (47.5) | 691 (37.9) | 129 (56.8) | 101 (39.9) | 3 (100.0) | 7 (4.6) | <0.001 |
| **Age (mean (SD))** | 72.82 (7.14) | 65.22 (13.71) | 71.39 (12.58) | 71.88 (8.81) | 69.34 (16.42) | 72.67 (2.89) | 62.66 (6.78) | <0.001 |
| **Sex = Female (%)** | 443 (49.3) | 65 (60.7) | 2403 (51.8) | 299 (53.8) | 223 (50.7) | 1 (16.7) | 229 (61.4) | 0.001 |
| ***APOE* e4 Carrier = Yes (%)** | 147 (68.1) | 0 | 1031 (55.8) | 109 (47.2) | 74 (58.7) | 2 (66.7) | 105 (54.4) | 0.001 |

The percentages presented in the table were based on participants with complete data for the corresponding variables. AD status, age, sex, and APOE e4 carrier status were compared using Fisher’s exact and two-tailed t-tests, where appropriate. Note that the comparison of APOE e4 carrier status was done without individuals from NIMH. Abbreviations: National Cell Repository for Alzheimer’s Disease (NCRAD), National Institute of Mental Health (NIMH), National Institute on Aging Late Onset Alzheimer’s Disease Family Study (NIA-LOAD), University of Miami (UM), University of Washington Families (UWF), Vanderbilt University (VU), Wisconsin Registry for Alzheimer’s Prevention (WRAP).
