# Supplementary material for: Dissecting Alzheimer's disease heritability across populations
Source: Alzheimers Dement. 2026 Mar 25;22(3):e71236. doi: 10.1002/alz.71236 (PMC13093350; doi:10.1002/alz.71236)
Supplement: Supplementary file 9 — Supporting Information [file ALZ-22-e71236-s003.docx]

Table S5 Basic demographics across studies for Non-Hispanic Blacks.

|  | **NCRAD** | **NIA-LOAD** | **UM** | **WRAP** | **p-value** |
| --- | --- | --- | --- | --- | --- |
| **n** | 42 | 192 | 39 | 13 |  |
| **AD = A (%)** | 3 (100.0) | 19 (29.7) | 10 (90.9) | 1 (16.7) | <0.001 |
| **Age (mean (SD))** | 71.50 (11.52) | 65.33 (12.75) | 77.08 (7.28) | 59.83 (6.24) | 0.006 |
| **Sex = Female (%)** | 20 (47.6) | 97 (50.5) | 20 (51.3) | 10 (76.9) | 0.297 |
| ***APOE* e4 Carrier = Yes (%)** | 10 (71.4) | 23 (34.8) | 11 (73.3) | 5 (71.4) | 0.005 |

The percentages presented in the table were based on participants with complete data for the corresponding variables. AD status, age, sex, and APOE e4 carrier status were compared using Fisher’s exact and two-tailed t-tests, where appropriate. Abbreviations: National Cell Repository for Alzheimer’s Disease (NCRAD), National Institute of Mental Health (NIMH), University of Miami (UM), Wisconsin Registry for Alzheimer’s Prevention (WRAP).
