# Supplementary material for: Dissecting Alzheimer's disease heritability across populations
Source: Alzheimers Dement. 2026 Mar 25;22(3):e71236. doi: 10.1002/alz.71236 (PMC13093350; doi:10.1002/alz.71236)
Supplement: Supplementary file 10 — Supporting Information [file ALZ-22-e71236-s010.docx]

Table S6 Basic demographics across studies for Dominicans.

|  | **EFIGA** | **NIA-LOAD** | **p-value** |
| --- | --- | --- | --- |
| **n** | 3603 | 385 |  |
| **AD = A (%)** | 547 (47.3) | 53 (44.5) | 0.630 |
| **Age (mean (SD))** | 68.97 (10.89) | 67.76 (12.12) | 0.248 |
| **Sex = Female (%)** | 1872 (52.0) | 190 (49.4) | 0.358 |
| **APOE e4 Carrier = Yes (%)** | 473 (39.2) | 36 (38.3) | 0.957 |

The percentages presented in the table were based on participants with complete data for the corresponding variables. AD status, age, sex, and APOE e4 carrier status were compared using Fisher’s exact and two-tailed t-tests, where appropriate. Abbreviations: Estudio Familiar de la Influencia Genetica en Alzheimer (EFIGA), National Institute on Aging Late Onset Alzheimer’s Disease Family Study (NIA-LOAD).
