# Supplementary material for: Dissecting Alzheimer's disease heritability across populations
Source: Alzheimers Dement. 2026 Mar 25;22(3):e71236. doi: 10.1002/alz.71236 (PMC13093350; doi:10.1002/alz.71236)
Supplement: Supplementary file 11 — Supporting Information [file ALZ-22-e71236-s007.docx]

Table S7 Effective sample sizes for S.A.G.E. and SOLAR analyses by group assignment.

|  | **S.A.G.E.** | | **SOLAR** | |
| --- | --- | --- | --- | --- |
|  | # pedigrees | # members | # pedigrees | # members |
| **Non-Hispanic White** | | | | |
| Model 1 | 414 | 2,602 | 411 | 2,608 |
| Model 2 | 414 | 2,349 | 403 | 2,354 |
| Model 3 | 414 | 2,602 | 411 | 2,608 |
| Model 4 | 414 | 2,349 | 403 | 2,354 |
| **Non-Hispanic Black** | | | | |
| Model 1 | 13 | 84 | 13 | 84 |
| Model 2 | 13 | 84 | 13 | 84 |
| Model 3 | 13 | 84 | 13 | 84 |
| Model 4 | 13 | 84 | 13 | 84 |
| **Dominican** | | | | |
| Model 1 | 100 | 1,271 | 103 | 1,275 |
| Model 2 | 100 | 1,231 | 103 | 1,235 |
| Model 3 | 100 | 1,271 | 103 | 1,275 |
| Model 4 | 100 | 1,231 | 103 | 1,235 |
| **Dutch Isolate** | | | | |
| Model 1 | 10 | 165 | 10 | 165 |
| Model 2 | 10 | 161 | 10 | 161 |
| Model 3 | 10 | 165 | 10 | 165 |
| Model 4 | 10 | 161 | 10 | 161 |

Covariates for adjustment in each model: Model 1, age, and sex; Model 2, age, sex, and *APOE* e4 carrier status; Model 3, age, sex, and study; Model 4, age, sex, *APOE* e4 carrier status, and study.
