# Supplementary material for: Dissecting Alzheimer's disease heritability across populations
Source: Alzheimers Dement. 2026 Mar 25;22(3):e71236. doi: 10.1002/alz.71236 (PMC13093350; doi:10.1002/alz.71236)
Supplement: Supplementary file 12 — Supporting Information [file ALZ-22-e71236-s015.docx]

Table S8 Distribution of original AD diagnoses by group assignment

|  | **Non-Hispanic White** | **Non-Hispanic Black** | **Dominican** | **Dutch Isolate** |
| --- | --- | --- | --- | --- |
| Total | 7,024 | 286 | 3,988 | 655 |
| Definite AD | 359 | 4 | 0 | 0 |
| Probable AD | 567 | 18 | 559 | 60 |
| Possible AD | 162 | 11 | 41 | 0 |
| No Dementia | 1,552 | 51 | 675 | 105 |
| Other Dementia | 95 | 2 | 15 | 0 |
| Family Reported AD | 466 | 25 | 112 | 23 |
| Family Reported No AD | 638 | 35 | 875 | 0 |
| Unknown | 3,185 | 140 | 1,711 | 467 |
