# Supplementary material for: Dissecting Alzheimer's disease heritability across populations
Source: Alzheimers Dement. 2026 Mar 25;22(3):e71236. doi: 10.1002/alz.71236 (PMC13093350; doi:10.1002/alz.71236)
Supplement: Supplementary file 13 — Supporting Information [file ALZ-22-e71236-s002.docx]

Table S9 S.A.G.E.-derived heritability estimates with cases defined as individuals having definite or probable AD diagnoses

|  | **Model1** | | | **Model2** | | | **Model3** | | | **Model4** | | |
| --- | --- | --- | --- | --- | --- | --- | --- | --- | --- | --- | --- | --- |
|  | $h^{2}$ | SE | p-value | $h^{2}$ | SE | p-value | $h^{2}$ | SE | p-value | $h^{2}$ | SE | p-value |
| **Non-Hispanic White** | 0.2718 | 0.0416 | < 1E-7 | 0.2718 | 0.0416 | < 1E-7 | 0.2368 | 0.0388 | < 1E-7 | 0.2046 | 0.0401 | < 1E-6 |
| **Non-Hispanic Black** | 0.3870 | 0.1882 | 0.0199 | 0.1245 | 0.2190 | 0.2849 | 0.3864 | 0.1875 | 0.0197 | 0.1242 | 0.2095 | 0.2766 |
| **Dominican** | 0.3112 | 0.0615 | < 1E-6 | 0.2904 | 0.0619 | < 1E-5 | 0.3112 | 0.0615 | < 1E-6 | 0.2900 | 0.0619 | < 1E-5 |

Covariates for adjustment in each model: Model1, age, and sex; Model2, age, sex, and *APOE* e4 carrier status; Model3, age, sex, and study; Model4, age, sex, *APOE* e4 carrier status, and study. Note that results for the Dutch Isolate group are not shown since the number of cases did not change compared to the definition in the main text. Abbreviation: standard error (SE).
