# Supplementary material for: Dissecting Alzheimer's disease heritability across populations
Source: Alzheimers Dement. 2026 Mar 25;22(3):e71236. doi: 10.1002/alz.71236 (PMC13093350; doi:10.1002/alz.71236)
Supplement: Supplementary file 14 — Supporting Information [file ALZ-22-e71236-s004.docx]

Table S10 SOLAR-derived heritability estimates with cases defined as individuals having definite or probable AD diagnoses

|  | **Model1** | | | **Model2** | | | **Model3** | | | **Model4** | | |
| --- | --- | --- | --- | --- | --- | --- | --- | --- | --- | --- | --- | --- |
|  | $h^{2}$ | SE | p-value | $h^{2}$ | SE | p-value | $h^{2}$ | SE | p-value | $h^{2}$ | SE | p-value |
| **Non-Hispanic White** | 0.4961 | 0.0791 | 1.18E-14 | 0.4583 | 0.0761 | 5.22E-11 | 0.4820 | 0.0739 | 3.48E-15 | 0.4585 | 0.0822 | 3.80E-12 |
| **Non-Hispanic Black** | 0.7446 | 0.5892 | 0.1102 | ND | | | 0.7468 | 0.597 | 0.1056 | ND | | |
| **Dominican** | 0.5357 | 0.1046 | 3.97E-11 | 0.4986 | 0.1027 | 1.55E-09 | 0.5349 | 0.1000 | 5.79E-11 | 0.5021 | 0.1070 | 1.37E-09 |

Covariates for adjustment in each model: Model1, age, and sex; Model2, age, sex, and APOE e4 carrier status; Model3, age, sex, and study; Model4, age, sex, APOE e4 carrier status, and study. Specifically, results for Model 2 and Model 4 within the non-Hispanic Black group are labeled as ND (no data) due to model convergence issues after incorporating APOE ε4 carrier status into the analysis. Note that results for the Dutch Isolate group are not shown since the number of cases did not change compared to the definition in the main text. Abbreviation: standard error (SE).
