# Supplementary material for: Dissecting Alzheimer's disease heritability across populations
Source: Alzheimers Dement. 2026 Mar 25;22(3):e71236. doi: 10.1002/alz.71236 (PMC13093350; doi:10.1002/alz.71236)
Supplement: Supplementary file 15 — Supporting Information [file ALZ-22-e71236-s012.docx]

Table S9 Heritability estimates for the non-Hispanic White group with cases defined as definite AD diagnoses only

|  | **Model1** | | | **Model2** | | | **Model3** | | | **Model4** | | |
| --- | --- | --- | --- | --- | --- | --- | --- | --- | --- | --- | --- | --- |
|  | $h^{2}$ | SE | p-value | $h^{2}$ | SE | p-value | $h^{2}$ | SE | p-value | $h^{2}$ | SE | p-value |
| **S.A.G.E.** | 0.5695 | 0.0453 | < 1E-7 | 0.5389 | 0.0509 | < 1E-7 | 0.5039 | 0.0470 | < 1E-7 | 0.4988 | 0.0509 | < 1E-7 |
| **SOLAR** | 0.8241 | 0.1006 | 1.97E-18 | 0.8548 | 0.1163 | 7.21E-15 | 0.7910 | 0.0995 | 1.71E-17 | 0.8059 | 0.1161 | 1.56E-13 |

Covariates for adjustment in each model: Model1, age, and sex; Model2, age, sex, and *APOE* e4 carrier status; Model3, age, sex, and study; Model4, age, sex, *APOE* e4 carrier status, and study. Abbreviation: standard error (SE).
